# Supplementary figures and images for: Search for morphological indicators that predict implantation by principal component analysis using images of blastocyst (part 1 of 2)
Source: PeerJ. 2022 May 16;10:e13441. doi: 10.7717/peerj.13441 (PMC9119295; doi:10.7717/peerj.13441)

**A**

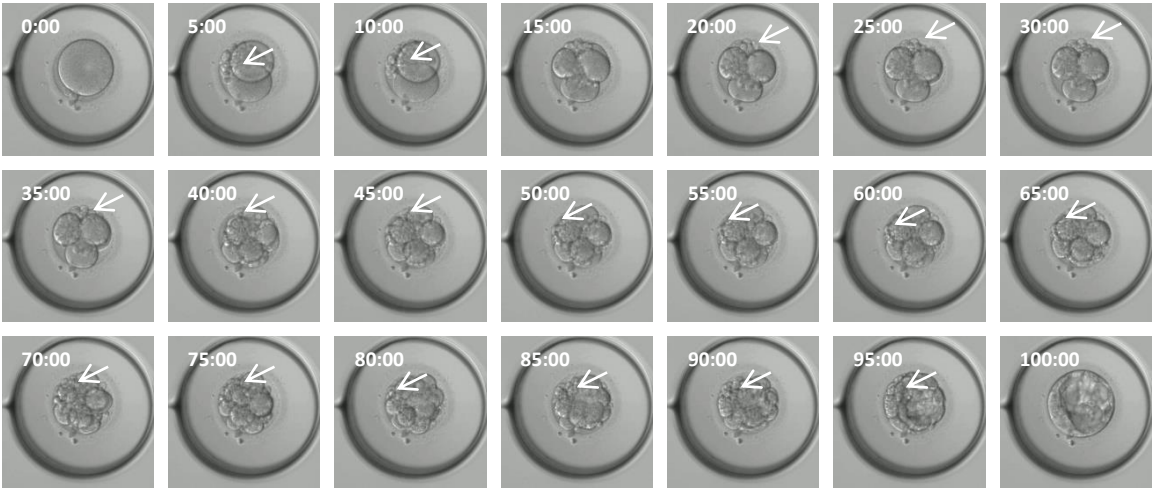

**B**

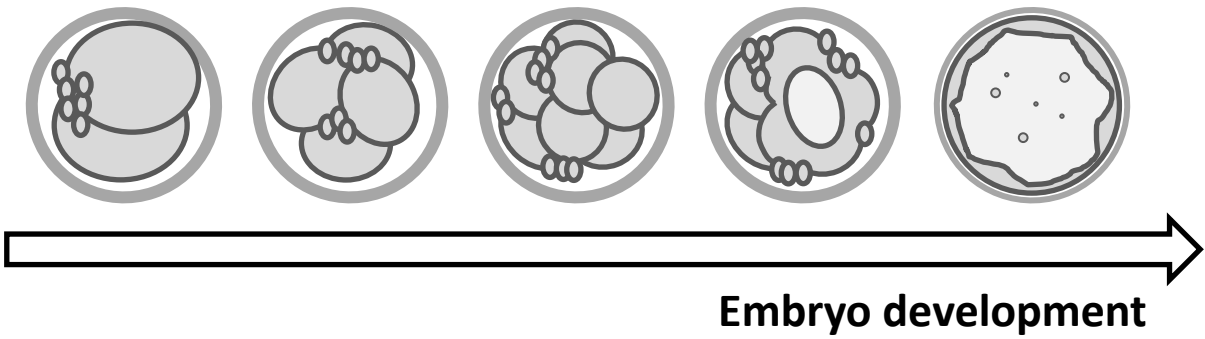

**Supplemental Fig. 1**

Supplement: Supplemental Information 2 — (A) A typical image showing the production of non-grainy blastocyst. (B) Illustrated process showing the production of non-grainy blastocyst. [file peerj-10-13441-s002.pdf]

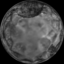

Supplement: Supplemental Information 4 [file peerj-10-13441-s004.zip › SI/0.png]

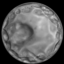

Supplement: Supplemental Information 4 [file peerj-10-13441-s004.zip › SI/1.png]

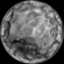

Supplement: Supplemental Information 4 [file peerj-10-13441-s004.zip › SI/10.png]

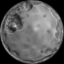

Supplement: Supplemental Information 4 [file peerj-10-13441-s004.zip › SI/100.png]

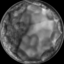

Supplement: Supplemental Information 4 [file peerj-10-13441-s004.zip › SI/101.png]

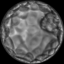

Supplement: Supplemental Information 4 [file peerj-10-13441-s004.zip › SI/102.png]

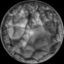

Supplement: Supplemental Information 4 [file peerj-10-13441-s004.zip › SI/103.png]

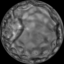

Supplement: Supplemental Information 4 [file peerj-10-13441-s004.zip › SI/104.png]

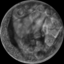

Supplement: Supplemental Information 4 [file peerj-10-13441-s004.zip › SI/105.png]

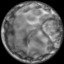

Supplement: Supplemental Information 4 [file peerj-10-13441-s004.zip › SI/106.png]

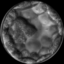

Supplement: Supplemental Information 4 [file peerj-10-13441-s004.zip › SI/107.png]

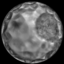

Supplement: Supplemental Information 4 [file peerj-10-13441-s004.zip › SI/108.png]

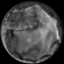

Supplement: Supplemental Information 4 [file peerj-10-13441-s004.zip › SI/109.png]

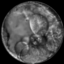

Supplement: Supplemental Information 4 [file peerj-10-13441-s004.zip › SI/11.png]

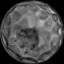

Supplement: Supplemental Information 4 [file peerj-10-13441-s004.zip › SI/110.png]

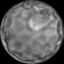

Supplement: Supplemental Information 4 [file peerj-10-13441-s004.zip › SI/111.png]

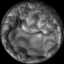

Supplement: Supplemental Information 4 [file peerj-10-13441-s004.zip › SI/112.png]

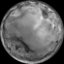

Supplement: Supplemental Information 4 [file peerj-10-13441-s004.zip › SI/113.png]

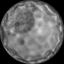

Supplement: Supplemental Information 4 [file peerj-10-13441-s004.zip › SI/114.png]

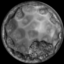

Supplement: Supplemental Information 4 [file peerj-10-13441-s004.zip › SI/115.png]

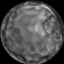

Supplement: Supplemental Information 4 [file peerj-10-13441-s004.zip › SI/116.png]

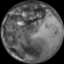

Supplement: Supplemental Information 4 [file peerj-10-13441-s004.zip › SI/117.png]

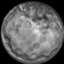

Supplement: Supplemental Information 4 [file peerj-10-13441-s004.zip › SI/118.png]

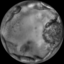

Supplement: Supplemental Information 4 [file peerj-10-13441-s004.zip › SI/119.png]

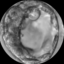

Supplement: Supplemental Information 4 [file peerj-10-13441-s004.zip › SI/12.png]

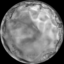

Supplement: Supplemental Information 4 [file peerj-10-13441-s004.zip › SI/120.png]

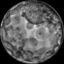

Supplement: Supplemental Information 4 [file peerj-10-13441-s004.zip › SI/121.png]

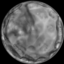

Supplement: Supplemental Information 4 [file peerj-10-13441-s004.zip › SI/122.png]

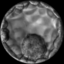

Supplement: Supplemental Information 4 [file peerj-10-13441-s004.zip › SI/123.png]

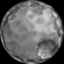

Supplement: Supplemental Information 4 [file peerj-10-13441-s004.zip › SI/124.png]

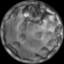

Supplement: Supplemental Information 4 [file peerj-10-13441-s004.zip › SI/125.png]

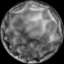

Supplement: Supplemental Information 4 [file peerj-10-13441-s004.zip › SI/126.png]

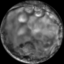

Supplement: Supplemental Information 4 [file peerj-10-13441-s004.zip › SI/127.png]

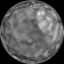

Supplement: Supplemental Information 4 [file peerj-10-13441-s004.zip › SI/128.png]

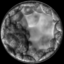

Supplement: Supplemental Information 4 [file peerj-10-13441-s004.zip › SI/129.png]

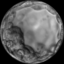

Supplement: Supplemental Information 4 [file peerj-10-13441-s004.zip › SI/13.png]

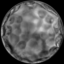

Supplement: Supplemental Information 4 [file peerj-10-13441-s004.zip › SI/130.png]

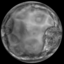

Supplement: Supplemental Information 4 [file peerj-10-13441-s004.zip › SI/131.png]

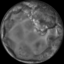

Supplement: Supplemental Information 4 [file peerj-10-13441-s004.zip › SI/132.png]

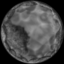

Supplement: Supplemental Information 4 [file peerj-10-13441-s004.zip › SI/133.png]

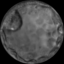

Supplement: Supplemental Information 4 [file peerj-10-13441-s004.zip › SI/134.png]

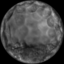

Supplement: Supplemental Information 4 [file peerj-10-13441-s004.zip › SI/135.png]

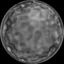

Supplement: Supplemental Information 4 [file peerj-10-13441-s004.zip › SI/136.png]

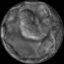

Supplement: Supplemental Information 4 [file peerj-10-13441-s004.zip › SI/137.png]

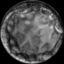

Supplement: Supplemental Information 4 [file peerj-10-13441-s004.zip › SI/14.png]

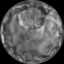

Supplement: Supplemental Information 4 [file peerj-10-13441-s004.zip › SI/140.png]

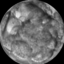

Supplement: Supplemental Information 4 [file peerj-10-13441-s004.zip › SI/141.png]

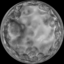

Supplement: Supplemental Information 4 [file peerj-10-13441-s004.zip › SI/142.png]

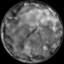

Supplement: Supplemental Information 4 [file peerj-10-13441-s004.zip › SI/143.png]

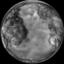

Supplement: Supplemental Information 4 [file peerj-10-13441-s004.zip › SI/144.png]

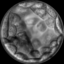

Supplement: Supplemental Information 4 [file peerj-10-13441-s004.zip › SI/145.png]

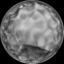

Supplement: Supplemental Information 4 [file peerj-10-13441-s004.zip › SI/146.png]

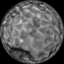

Supplement: Supplemental Information 4 [file peerj-10-13441-s004.zip › SI/147.png]

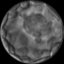

Supplement: Supplemental Information 4 [file peerj-10-13441-s004.zip › SI/148.png]

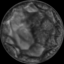

Supplement: Supplemental Information 4 [file peerj-10-13441-s004.zip › SI/149.png]

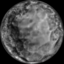

Supplement: Supplemental Information 4 [file peerj-10-13441-s004.zip › SI/15.png]

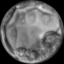

Supplement: Supplemental Information 4 [file peerj-10-13441-s004.zip › SI/150.png]

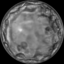

Supplement: Supplemental Information 4 [file peerj-10-13441-s004.zip › SI/151.png]

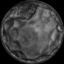

Supplement: Supplemental Information 4 [file peerj-10-13441-s004.zip › SI/152.png]

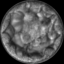

Supplement: Supplemental Information 4 [file peerj-10-13441-s004.zip › SI/153.png]

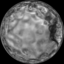

Supplement: Supplemental Information 4 [file peerj-10-13441-s004.zip › SI/154.png]

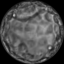

Supplement: Supplemental Information 4 [file peerj-10-13441-s004.zip › SI/155.png]

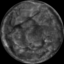

Supplement: Supplemental Information 4 [file peerj-10-13441-s004.zip › SI/156.png]

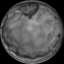

Supplement: Supplemental Information 4 [file peerj-10-13441-s004.zip › SI/157.png]

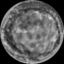

Supplement: Supplemental Information 4 [file peerj-10-13441-s004.zip › SI/158.png]

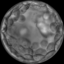

Supplement: Supplemental Information 4 [file peerj-10-13441-s004.zip › SI/159.png]

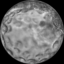

Supplement: Supplemental Information 4 [file peerj-10-13441-s004.zip › SI/16.png]

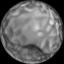

Supplement: Supplemental Information 4 [file peerj-10-13441-s004.zip › SI/160.png]

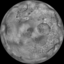

Supplement: Supplemental Information 4 [file peerj-10-13441-s004.zip › SI/161.png]

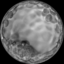

Supplement: Supplemental Information 4 [file peerj-10-13441-s004.zip › SI/162.png]

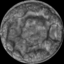

Supplement: Supplemental Information 4 [file peerj-10-13441-s004.zip › SI/163.png]

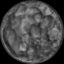

Supplement: Supplemental Information 4 [file peerj-10-13441-s004.zip › SI/164.png]

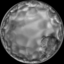

Supplement: Supplemental Information 4 [file peerj-10-13441-s004.zip › SI/165.png]

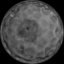

Supplement: Supplemental Information 4 [file peerj-10-13441-s004.zip › SI/166.png]

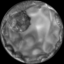

Supplement: Supplemental Information 4 [file peerj-10-13441-s004.zip › SI/167.png]

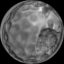

Supplement: Supplemental Information 4 [file peerj-10-13441-s004.zip › SI/168.png]

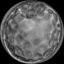

Supplement: Supplemental Information 4 [file peerj-10-13441-s004.zip › SI/169.png]

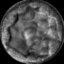

Supplement: Supplemental Information 4 [file peerj-10-13441-s004.zip › SI/17.png]

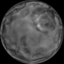

Supplement: Supplemental Information 4 [file peerj-10-13441-s004.zip › SI/170.png]

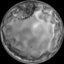

Supplement: Supplemental Information 4 [file peerj-10-13441-s004.zip › SI/171.png]

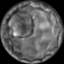

Supplement: Supplemental Information 4 [file peerj-10-13441-s004.zip › SI/172.png]

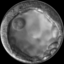

Supplement: Supplemental Information 4 [file peerj-10-13441-s004.zip › SI/173.png]

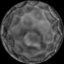

Supplement: Supplemental Information 4 [file peerj-10-13441-s004.zip › SI/174.png]

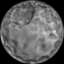

Supplement: Supplemental Information 4 [file peerj-10-13441-s004.zip › SI/175.png]

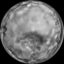

Supplement: Supplemental Information 4 [file peerj-10-13441-s004.zip › SI/176.png]

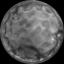

Supplement: Supplemental Information 4 [file peerj-10-13441-s004.zip › SI/177.png]

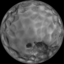

Supplement: Supplemental Information 4 [file peerj-10-13441-s004.zip › SI/178.png]

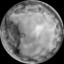

Supplement: Supplemental Information 4 [file peerj-10-13441-s004.zip › SI/179.png]

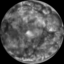

Supplement: Supplemental Information 4 [file peerj-10-13441-s004.zip › SI/18.png]

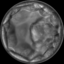

Supplement: Supplemental Information 4 [file peerj-10-13441-s004.zip › SI/180.png]

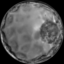

Supplement: Supplemental Information 4 [file peerj-10-13441-s004.zip › SI/181.png]

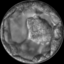

Supplement: Supplemental Information 4 [file peerj-10-13441-s004.zip › SI/182.png]

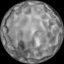

Supplement: Supplemental Information 4 [file peerj-10-13441-s004.zip › SI/183.png]

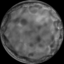

Supplement: Supplemental Information 4 [file peerj-10-13441-s004.zip › SI/184.png]

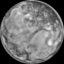

Supplement: Supplemental Information 4 [file peerj-10-13441-s004.zip › SI/185.png]

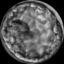

Supplement: Supplemental Information 4 [file peerj-10-13441-s004.zip › SI/186.png]

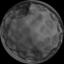

Supplement: Supplemental Information 4 [file peerj-10-13441-s004.zip › SI/187.png]

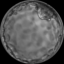

Supplement: Supplemental Information 4 [file peerj-10-13441-s004.zip › SI/188.png]

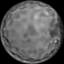

Supplement: Supplemental Information 4 [file peerj-10-13441-s004.zip › SI/189.png]
