# Supplementary figures and images for: Search for morphological indicators that predict implantation by principal component analysis using images of blastocyst (part 2 of 2)
Source: PeerJ. 2022 May 16;10:e13441. doi: 10.7717/peerj.13441 (PMC9119295; doi:10.7717/peerj.13441)

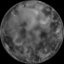

Supplement: Supplemental Information 4 [file peerj-10-13441-s004.zip › SI/19.png]

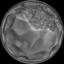

Supplement: Supplemental Information 4 [file peerj-10-13441-s004.zip › SI/190.png]

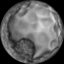

Supplement: Supplemental Information 4 [file peerj-10-13441-s004.zip › SI/191.png]

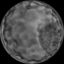

Supplement: Supplemental Information 4 [file peerj-10-13441-s004.zip › SI/192.png]

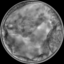

Supplement: Supplemental Information 4 [file peerj-10-13441-s004.zip › SI/193.png]

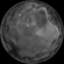

Supplement: Supplemental Information 4 [file peerj-10-13441-s004.zip › SI/194.png]

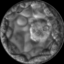

Supplement: Supplemental Information 4 [file peerj-10-13441-s004.zip › SI/195.png]

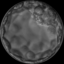

Supplement: Supplemental Information 4 [file peerj-10-13441-s004.zip › SI/196.png]

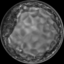

Supplement: Supplemental Information 4 [file peerj-10-13441-s004.zip › SI/197.png]

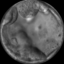

Supplement: Supplemental Information 4 [file peerj-10-13441-s004.zip › SI/198.png]

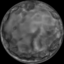

Supplement: Supplemental Information 4 [file peerj-10-13441-s004.zip › SI/199.png]

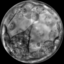

Supplement: Supplemental Information 4 [file peerj-10-13441-s004.zip › SI/2.png]

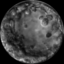

Supplement: Supplemental Information 4 [file peerj-10-13441-s004.zip › SI/20.png]

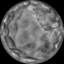

Supplement: Supplemental Information 4 [file peerj-10-13441-s004.zip › SI/200.png]

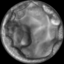

Supplement: Supplemental Information 4 [file peerj-10-13441-s004.zip › SI/201.png]

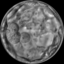

Supplement: Supplemental Information 4 [file peerj-10-13441-s004.zip › SI/202.png]

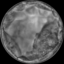

Supplement: Supplemental Information 4 [file peerj-10-13441-s004.zip › SI/203.png]

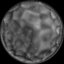

Supplement: Supplemental Information 4 [file peerj-10-13441-s004.zip › SI/204.png]

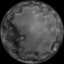

Supplement: Supplemental Information 4 [file peerj-10-13441-s004.zip › SI/205.png]

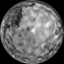

Supplement: Supplemental Information 4 [file peerj-10-13441-s004.zip › SI/206.png]

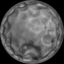

Supplement: Supplemental Information 4 [file peerj-10-13441-s004.zip › SI/207.png]

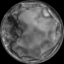

Supplement: Supplemental Information 4 [file peerj-10-13441-s004.zip › SI/208.png]

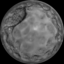

Supplement: Supplemental Information 4 [file peerj-10-13441-s004.zip › SI/209.png]

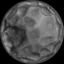

Supplement: Supplemental Information 4 [file peerj-10-13441-s004.zip › SI/21.png]

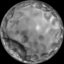

Supplement: Supplemental Information 4 [file peerj-10-13441-s004.zip › SI/210.png]

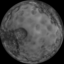

Supplement: Supplemental Information 4 [file peerj-10-13441-s004.zip › SI/211.png]

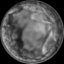

Supplement: Supplemental Information 4 [file peerj-10-13441-s004.zip › SI/212.png]

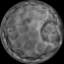

Supplement: Supplemental Information 4 [file peerj-10-13441-s004.zip › SI/213.png]

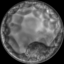

Supplement: Supplemental Information 4 [file peerj-10-13441-s004.zip › SI/214.png]

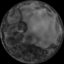

Supplement: Supplemental Information 4 [file peerj-10-13441-s004.zip › SI/215.png]

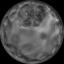

Supplement: Supplemental Information 4 [file peerj-10-13441-s004.zip › SI/216.png]

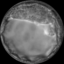

Supplement: Supplemental Information 4 [file peerj-10-13441-s004.zip › SI/217.png]

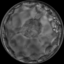

Supplement: Supplemental Information 4 [file peerj-10-13441-s004.zip › SI/218.png]

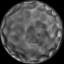

Supplement: Supplemental Information 4 [file peerj-10-13441-s004.zip › SI/219.png]

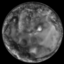

Supplement: Supplemental Information 4 [file peerj-10-13441-s004.zip › SI/22.png]

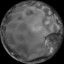

Supplement: Supplemental Information 4 [file peerj-10-13441-s004.zip › SI/220.png]

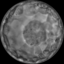

Supplement: Supplemental Information 4 [file peerj-10-13441-s004.zip › SI/221.png]

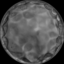

Supplement: Supplemental Information 4 [file peerj-10-13441-s004.zip › SI/222.png]

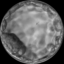

Supplement: Supplemental Information 4 [file peerj-10-13441-s004.zip › SI/223.png]

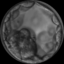

Supplement: Supplemental Information 4 [file peerj-10-13441-s004.zip › SI/224.png]

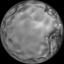

Supplement: Supplemental Information 4 [file peerj-10-13441-s004.zip › SI/225.png]

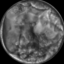

Supplement: Supplemental Information 4 [file peerj-10-13441-s004.zip › SI/226.png]

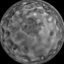

Supplement: Supplemental Information 4 [file peerj-10-13441-s004.zip › SI/227.png]

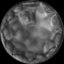

Supplement: Supplemental Information 4 [file peerj-10-13441-s004.zip › SI/228.png]

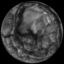

Supplement: Supplemental Information 4 [file peerj-10-13441-s004.zip › SI/229.png]

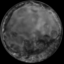

Supplement: Supplemental Information 4 [file peerj-10-13441-s004.zip › SI/23.png]

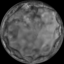

Supplement: Supplemental Information 4 [file peerj-10-13441-s004.zip › SI/230.png]

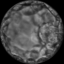

Supplement: Supplemental Information 4 [file peerj-10-13441-s004.zip › SI/231.png]

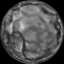

Supplement: Supplemental Information 4 [file peerj-10-13441-s004.zip › SI/232.png]

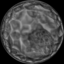

Supplement: Supplemental Information 4 [file peerj-10-13441-s004.zip › SI/233.png]

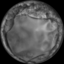

Supplement: Supplemental Information 4 [file peerj-10-13441-s004.zip › SI/24.png]

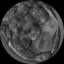

Supplement: Supplemental Information 4 [file peerj-10-13441-s004.zip › SI/25.png]

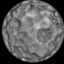

Supplement: Supplemental Information 4 [file peerj-10-13441-s004.zip › SI/26.png]

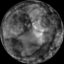

Supplement: Supplemental Information 4 [file peerj-10-13441-s004.zip › SI/27.png]

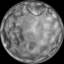

Supplement: Supplemental Information 4 [file peerj-10-13441-s004.zip › SI/28.png]

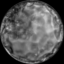

Supplement: Supplemental Information 4 [file peerj-10-13441-s004.zip › SI/29.png]

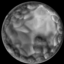

Supplement: Supplemental Information 4 [file peerj-10-13441-s004.zip › SI/3.png]

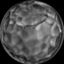

Supplement: Supplemental Information 4 [file peerj-10-13441-s004.zip › SI/30.png]

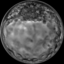

Supplement: Supplemental Information 4 [file peerj-10-13441-s004.zip › SI/31.png]

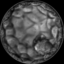

Supplement: Supplemental Information 4 [file peerj-10-13441-s004.zip › SI/32.png]

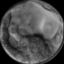

Supplement: Supplemental Information 4 [file peerj-10-13441-s004.zip › SI/33.png]

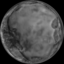

Supplement: Supplemental Information 4 [file peerj-10-13441-s004.zip › SI/34.png]

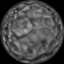

Supplement: Supplemental Information 4 [file peerj-10-13441-s004.zip › SI/35.png]

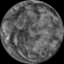

Supplement: Supplemental Information 4 [file peerj-10-13441-s004.zip › SI/36.png]

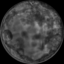

Supplement: Supplemental Information 4 [file peerj-10-13441-s004.zip › SI/37.png]

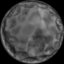

Supplement: Supplemental Information 4 [file peerj-10-13441-s004.zip › SI/38.png]

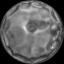

Supplement: Supplemental Information 4 [file peerj-10-13441-s004.zip › SI/39.png]

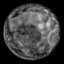

Supplement: Supplemental Information 4 [file peerj-10-13441-s004.zip › SI/4.png]

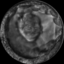

Supplement: Supplemental Information 4 [file peerj-10-13441-s004.zip › SI/40.png]

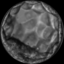

Supplement: Supplemental Information 4 [file peerj-10-13441-s004.zip › SI/41.png]

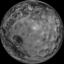

Supplement: Supplemental Information 4 [file peerj-10-13441-s004.zip › SI/42.png]

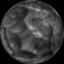

Supplement: Supplemental Information 4 [file peerj-10-13441-s004.zip › SI/43.png]

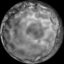

Supplement: Supplemental Information 4 [file peerj-10-13441-s004.zip › SI/44.png]

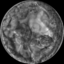

Supplement: Supplemental Information 4 [file peerj-10-13441-s004.zip › SI/45.png]

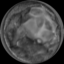

Supplement: Supplemental Information 4 [file peerj-10-13441-s004.zip › SI/46.png]

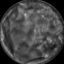

Supplement: Supplemental Information 4 [file peerj-10-13441-s004.zip › SI/47.png]

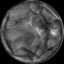

Supplement: Supplemental Information 4 [file peerj-10-13441-s004.zip › SI/48.png]

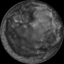

Supplement: Supplemental Information 4 [file peerj-10-13441-s004.zip › SI/49.png]

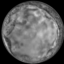

Supplement: Supplemental Information 4 [file peerj-10-13441-s004.zip › SI/5.png]

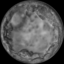

Supplement: Supplemental Information 4 [file peerj-10-13441-s004.zip › SI/50.png]

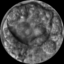

Supplement: Supplemental Information 4 [file peerj-10-13441-s004.zip › SI/51.png]

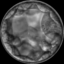

Supplement: Supplemental Information 4 [file peerj-10-13441-s004.zip › SI/52.png]

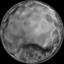

Supplement: Supplemental Information 4 [file peerj-10-13441-s004.zip › SI/53.png]

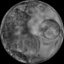

Supplement: Supplemental Information 4 [file peerj-10-13441-s004.zip › SI/54.png]

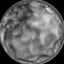

Supplement: Supplemental Information 4 [file peerj-10-13441-s004.zip › SI/55.png]

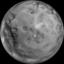

Supplement: Supplemental Information 4 [file peerj-10-13441-s004.zip › SI/56.png]

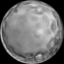

Supplement: Supplemental Information 4 [file peerj-10-13441-s004.zip › SI/57.png]

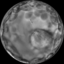

Supplement: Supplemental Information 4 [file peerj-10-13441-s004.zip › SI/58.png]

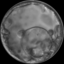

Supplement: Supplemental Information 4 [file peerj-10-13441-s004.zip › SI/59.png]

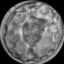

Supplement: Supplemental Information 4 [file peerj-10-13441-s004.zip › SI/6.png]

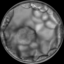

Supplement: Supplemental Information 4 [file peerj-10-13441-s004.zip › SI/60.png]

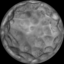

Supplement: Supplemental Information 4 [file peerj-10-13441-s004.zip › SI/61.png]

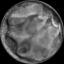

Supplement: Supplemental Information 4 [file peerj-10-13441-s004.zip › SI/62.png]

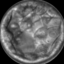

Supplement: Supplemental Information 4 [file peerj-10-13441-s004.zip › SI/63.png]

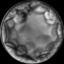

Supplement: Supplemental Information 4 [file peerj-10-13441-s004.zip › SI/64.png]

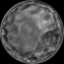

Supplement: Supplemental Information 4 [file peerj-10-13441-s004.zip › SI/65.png]

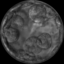

Supplement: Supplemental Information 4 [file peerj-10-13441-s004.zip › SI/66.png]

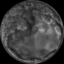

Supplement: Supplemental Information 4 [file peerj-10-13441-s004.zip › SI/67.png]

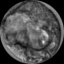

Supplement: Supplemental Information 4 [file peerj-10-13441-s004.zip › SI/68.png]
